# Knowledge, practice and perceived barriers towards chemotherapy induced nausea and vomiting in prophylaxis guideline adherence among nurses in oncology units at selected hospitals, in Addis Ababa, Ethiopia, a cross-sectional study

**DOI:** 10.1186/s12912-022-01009-7

**Published:** 2022-08-11

**Authors:** Deneke Gebre, Rajalakshmi Murugan, Ketema Bizuwork, Teshome Habte Wurjine

**Affiliations:** grid.7123.7 0000 0001 1250 5688 AAU, CHS, School of Nursing and Midwifery, P.O. Box: 5657, Addis Ababa, Ethiopia

**Keywords:** Chemotherapy-induced nausea and vomiting, Nurses’ knowledge, Guidelines, Adherence

## Abstract

**Background:**

Chemotherapy-induced emesis can be prevented by the use of recommended guidelines for antiemetic regimens but a research study indicates that in Ethiopia the use of standard antiemetic drug guidelines is very limited.

**Objectives:**

To assess knowledge, practice, and perceived barriers towards chemotherapy-induced nausea and vomiting in prophylaxis guideline adherence among nurses in oncology units.

**Methods:**

A cross-sectional study design was conducted among 81 oncology nurses selected in the two public hospitals of Addis Ababa, from March 1 to 30, 2020. The study participants were selected by using the population census method from the source population of nurses in oncology units. Data has collected by using semi-structured questionnaires with the self-administrated method. Data were analyzed by using Statistical Package for the Social Sciences software version 24. Descriptive statistics and logistic regression including bivariate and multivariate were conducted to examine the association between independent and outcome variables. The level of significance was determined at a *p*-value < 0.05 and a 95% confidence interval.

**Result:**

Seventy-nine nurses participated with a 96% of response rate. All participants were aged greater than 24 with a mean age of 28.8 ± 6 years and nearly two-thirds of the respondents (60.8%) were females. Nurses were not trained in chemotherapy-induced nausea and vomiting management shows 54.4%. nurses’ knowledge of chemotherapy-induced nausea and vomiting prophylaxis Guidelines was 78.5%. The means score of oncology nurses’ practice toward guideline recommendation was 41.8%. Knowledge of nurses associated with the use of chemotherapy-induced nausea and vomiting prophylaxis guideline recommendations working in the outpatient department, inpatient ward, and chemotherapy administration unit has a significant association with chemotherapy-induced nausea and vomiting management knowledge. In the multiple logistic regression analysis, nurses who have trained for chemotherapy-induced nausea and vomiting management were 1.64-fold more aware than those who were not trained.

**Conclusion:**

The study reveals that nurses working in the oncology unit of the study hospitals have a poor practice of Chemotherapy-Induced Nausea and Vomiting. Therefore, recommended providing Training for the Nurses working in the oncology unit and encourage them to apply standard guidelines.

## Introduction

Worldwide, more than 12 million individuals are newly diagnosed with cancer disease annually of these 8.2 million cancer deaths indicated, and from this 65% occurred in low-income countries [1]. Cancer Patients experience a considerable number of symptoms during the course of their disease [2], of these symptoms, chemotherapy-induced nausea and vomiting is one of the most reported, and it increases cancer burden on patients with dehydration, electrolyte imbalance and alters the metabolic activity of the body [3]. Chemotherapy-induced nausea and vomiting is a collective term used to describe the presentation of nausea or vomiting, or a combination of both symptoms associated with the administration of cytotoxic chemotherapy [4, 5].

Knowledge of Nurses to triage patient problems and assist in the evaluation of symptoms and initiation of interventions, subjective and objective data, including information about the last chemotherapy treatment, guide the nurse in determining of patient’s disposition and treatment [6, 7]. Oncology nursing professionals play a key role in the care of patients receiving chemotherapy. However, to do so, they require access to the most recent clinical information and guidance, the latest developments in chemotherapy induced nausea and vomiting therapy, and expanded knowledge of chemotherapy induced nausea and vomiting. Oncology nurses have assisted in the development of chemotherapy induced nausea and vomiting prophylaxis guidelines for the use of antiemetics particularly the 5-hydroxytryptamine–receptor antagonists, these guidelines outline the optimal use and safe delivery of antiemetic drugs and have proved to be an effective means of cost containment [8, 9].

In general, the guidelines recommend prescribing a Neurokinin-1 (NK-1) receptor antagonist along with a Serotonin5-hydroxytrypitamin (5-HT3) receptor antagonist and dexamethasone for prevention of chemotherapy induced nausea and vomiting in patients receiving high emetic chemotherapy, and a 5-HT3 receptor antagonist and dexamethasone in patients receiving moderately emetic chemotherapy [10].

Chemotherapy-induced nausea and vomiting (CINV) is a common problem occurring in the absence of antiemetic drugs in up to 99% of patients treated with highly emetogenic chemotherapy (HEC) and in 30%to 90%of those receiving moderately emetogenic chemotherapy [11]. Several institutional guidelines on the management of chemotherapy-induced nausea and vomiting are released and updated regularly, for example, the National Comprehensive Cancer Network (NCCN), oncology nursing society (ONS), American society of clinical oncology (ASCO) and the multinational association of supportive care in cancer (MASCC). Despite the existence of guidelines, Chemotherapy-induced nausea and vomiting remain prevalent and the guidelines are not fully implemented in clinical practice [12]. This results in under treatment of Chemotherapy-induced nausea and vomiting, which may lead to delaying or discontinuing chemotherapy treatment and lead to a reduced quality of life [13]. The side-effect of Chemotherapy can result in significant morbidity and can negatively affect the quality of life. In most cases chemotherapy-induced nausea and vomiting (CINV) can be prevented with the use of antiemetic regimens and recommended guidelines and it can result in significant morbidity and can negatively affect the quality of life. In Ethiopia many patients were suffering from CINV, in our hospitals adherence of antiemetic regimens recommended guidelines are available, but many instances adherence to the guidelines are limited by the nurses who are working in oncology unit. Therefore, this research to assess antiemetic guideline awareness and practice patterns of antiemetic use, determine adherence to the recommendations. A research study indicates that in many instances adherence to antiemetic drug guidelines is very limited. Nurses who are working in the oncology unit, and Oncology nurses who are part of a multidisciplinary team, can promote the use of appropriate antiemetic prophylaxis. Therefore, those nurses were surveyed to assess antiemetic guideline awareness and practice patterns of antiemetic use, and determine adherence to the recommended guidelines [14, 15].

A very limited research studies conducted in Ethiopia related to knowledge, skill, and to avoiding or minimizing barriers of nurses to manage chemotherapy-induced nausea, and vomiting, so this finding may improve the quality-of-service delivery in the clinical area. Specifically, this finding identifies the gap in knowledge, practice, and perceived barriers to managing Chemotherapy-induced nausea and vomiting using guidelines are very important to make institutions service evidence-based practice; to utilize antiemetic guideline for the successful prevention and management of chemotherapy-induced nausea and vomiting.

### Conceptual frame work

**Fig. 1:**
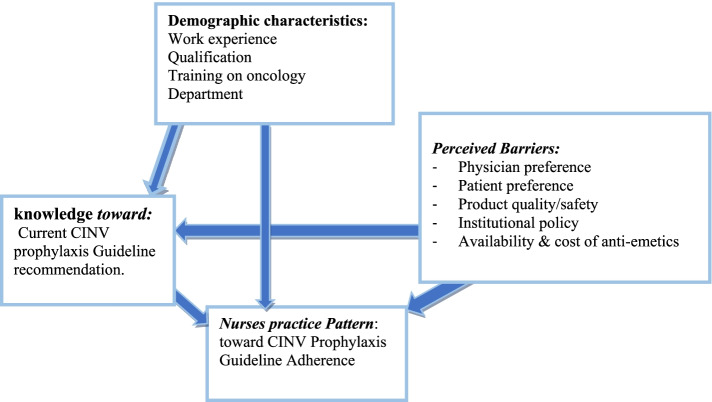
Conceptual frame-work on nurses’ knowledge practice pattern, and perceived barrier of chemotherapy induced nausea and vomiting toward prophylaxis guideline adherence nurses in oncology units of selected hospitals in Addis Ababa, Ethiopia

This conceptual framework shows the relationship between the dependent and independent variables of nurses’ knowledge, practice pattern, and perceived barrier of chemotherapy-induced nausea and vomiting toward prophylaxis guideline adherence as depicted in Fig. 1 below. This framework was developed after reviewing different literature and adopted towards the sociodemographic status of the study population [15–18].

## Methods and materials

### Study area

The study was conducted at selected public hospitals of Addis Ababa city Administration, Ethiopia. Addis Ababa is the largest and the most populated capital city of Ethiopia. The Addis Ababa metropolitan area with a population was estimated to be around 5,00,6000 people in 2021, This capital city holds 527 square kilometers of area in Ethiopia. The population density is estimated to be near 5,165 individuals per square kilometer available. Based on the 2020 population enumeration annual growth rate is 4.42% [19]. The city has 15 public hospitals from this only two hospitals have oncology unit and purposively selected to be included in this study. Those hospitals are: Tikur Anbessa Specialized Hospital and St. Pauls’ Hospital Millennium Medical College.

1. Tikur Anbessa Specialized Hospital: is one of tertiary and the largest hospital in the country with 850 beds hospital and currently 800 nurses working in this hospital and 56 nurses working in oncology units and the hospital annual report reveal that 370,000–400,000 patients treated per year [19].
2. St. Pauls’ Hospital Millennium Medical College: is a teaching and general hospital with 392 beds and 380 nurses providing service in the hospital, from these 26 nurses working in the oncology units [14, 19]. These two hospitals have an oncology service for chemotherapy.

### Study design and study period

An institution based-quantitative cross-sectional study design was conducted among nurses working in the selected public hospitals from March 1to 30, 2020.

### Study population and eligibility criteria

The study population was nurses working at oncology units of selected hospitals in Addis Ababa who fulfilled the eligibility criteria.

The Nurses who were currently working in the oncology ward, oncology OPD, and Day-care center for Chemotherapy Administration at selected hospitals during the data collection period and nurses who had more than six months of work experience in the chemotherapy administration unit were included in this study, whereas nurses who were off duty and less than six months work experience in the oncology unit were excluded from the study.

#### Sample size determination and sampling technique

The survey was applied to the two selected public hospital nurses who were working in the oncology unit assessed by using the census method [7, 9, 15]. This is due to the complete survey used for all nurses working in the oncology unit of the study hospitals. The total population of study participants currently working in the oncology unit of the study hospitals was 81 nurses which are smaller than what we found using simple population proportion formula. Finally, the entire population was included in this study. The study area was selected by using the purposive sampling method, out of fifteen governmental hospitals in Addis Ababa only two hospitals were selected. The study population selected by the population census formula and all nurses who were working in the oncology units and meet the inclusion criteria were included.

#### Data Collection Instrument and procedures

The data collection instrument was adapted from different literature related to the study problem and some modifications were applied to suit the study setting [15, 17]. The data collection tool consists of four sections: part one includes demographic questions, part two includes nurses’ knowledge of guideline recommendations, part- three concerned with nurses’ practice pattern toward current Chemotherapy-induced nausea and vomiting prophylaxis Guideline adherence, and part four about the nurses’ perceived barriers toward adherence to the guideline. The questionnaires were pre-tested two weeks before the actual data collection period, to check the quality of data and clarity. Based on the finding the necessary amendment was undertaken. Six BSc nurses were used as data collectors and two MSc nurses were supervisors involved in the data collection process. Data collectors explained the objective and purpose of the study. From the study participants, oral consent was obtained before the administration of the questionnaire. The one-day training was provided for data collectors and supervisors on the data collection instrument (on the clarity, and reliability of methods and materials).

### Study variables

#### Dependent variable

Nurses’ knowledge and practice Pattern toward antiemetic guideline Adherence.

#### Independent variables

- Demographic Characters: Age, Gender, work experience in chemotherapy administration in year, level of academic qualification, Work setting (OPD, Ward) and Oncology related training (supportive care or Inservice training).
- Perceived Barriers: physician interference, patient preferences, product cost, perceived compliance, products unavailability and products safety.

### Operational definitions and definition of terms

- Adequate knowledge: The nurse who answers the questionnaires, scored mean, and above considered as having adequate knowledge.
- In-Adequate knowledge: The nurse who answers the questionnaires, scored below the mean, considered as having in-adequate knowledge.
- Good Guideline-Adherence: Among the three international chemotherapy-induced nausea and vomiting guidelines recommendations, the nurses who report that they were utilizing at least one of the guideline recommendations for prophylaxis of chemotherapy induced nausea and vomiting and respond above the mean score value of practice questionaries said to be adherent.
- Guideline Non –Adherence: Among the three international guidelines recommendations, the nurses who report that they do not utilizing any of the guideline’s recommendation for prophylaxis of Chemotherapy induced nausea and vomiting, and respond below the mean score value of practice questionaries said to be non- adherent.
- Perceived Barriers: The Nurses mention the reason or condition, which was affect their utilization of international guideline recommended for chemotherapy-induced nausea and vomiting.

### Quality control

The training was provided for data collectors in regard to methods, materials, and how the data collection procedures were proceeding. Pretest was done with 5% of the sample population (nurses) from Hawassa referral hospital. Content validity and reliability were done to enrich the data collection tool. Based on the finding the necessary amendment was undertaken before proceeding to the actual data collection process.

### Data processing and analysis

The collected data were checked for completeness, edited, and entered into Epi data version 4.2.2 and exported to SPSS version 24 for analysis, Descriptive result was presented with frequencies, means, and standard deviations from the demographic data. Chi-square analysis was used to compare the response rate of Nurses’ confidence toward their knowledge of emetogenicity of chemotherapy regimen or trends in response based on demographic characteristics. Bivariate analysis was used to see the association of independent and dependent variables. A logistic regression model was employed to control confounding variables, and some of the statistical tests like odds ratio (crude & adjusted) was applied.

A weighted Rank score was calculated for the response regarding the barriers in preventing and managing Chemotherapy-induced nausea and vomiting in their practices. Respondents were asked to rank the top three responses from a list of nine perceived barriers; which were already listed on questionnaires, the top-ranked response got three points, the second received two points, and the third received one point. Finally adding all the responses of the Participant, the response which gets a higher score was the major perceived barrier of nurses which makes the nurses poorly adherent to Chemotherapy Induced nausea and vomiting (CINV) guideline.

## Result

### Socio-demographic and professional characteristics

A total of 79 nurses who were working in oncology unity of two selected public hospitals were assessed with a 96% of response rate. All participants were aged greater than 24 with a mean age of 28.8 ± 6 years and two-thirds of the respondents were female (60.8%).

Most of the respondents have a BSc degree (87.3%), followed by (12.7%) of them were MSc holders, and the remaining 3.8% of them were diploma holders as shown in Table 1 below, Of the total 57% of them haven’t an oncology certificate and 54.4% of the nurses don’t have any training in regard to chemotherapy-induced nausea and vomiting management training. 83.5% of the nurses have less than 5 years of clinical experience in oncology. 34.8% of them work in the chemotherapy administration unit and the others were in the oncology ward and outpatient department (29.1%and 35.4%) respectively. Only 2.5% of nurses were working as nurse managers while the majority (91%) of them served as staff nurses.

**Table 1:** Knowledge, practice and perceived barriers towards chemotherapy induced nausea and vomiting in prophylaxis guideline adherence of oncology unit nurses in the study hospitals,Addis Ababa, Ethiopia (*n* = 79)

*Factor*	*Category*	*Frequency(n)*	*Percent (%)*
Age(years)	20–30	51	64.6%
	31–40	26	32.9%
	41–50	2	2.5%
	Above50	0	0%
Sex	Male	31	39.2%
	Female	48	60.8%
Working department	OPD	28	35.4%
	Ward	23	29.1%
	Chemo administration unit	28	35.4%
Educational level	Diploma	3	3.8%
	BSc	66	87.3%
	MSc	10	12.7%
Are you certified in oncology nursing?	Yes	34	43%
	No	45	57%
Have you trained for CINV management?	Yes	36	45.6%
	No	43	54.4%
Nursing experience in oncology unit	Less than 5 years	66	83.5%
	6–10	10	12.7%
	11–15	3	3.8%
	More than 16 years	0	0%
Position in your department	Staff nurse	72	91.1%
	Nurse Supervisor	5	6.3%
	Nurse manager (matron)	2	2.6%

### Knowledge toward guideline recommendation

The oncology nurses assessed with 5 items for their level of knowledge towards the application of recommended guidelines are above 61% as depicted in Table 2 below. The rest 7 items scored below the mean score, more than 78% of the nurses have good knowledge toward considering emetogenic potentials of different chemotherapy when choosing anti-emetic for prophylaxis of chemotherapy-induced nausea and vomiting, whereas about 63% of the nurses are not confident in their knowledge of emetogenic classification of different type of chemotherapy.

**Table 2:** Knowledge of guideline recommendation, practice pattern and, perceived barriers toward chemotherapy induced nausea and vomiting prophylaxis guideline adherence among nurses working in the study hospitals. (*n* = 79)

Question	Yes (n)	percent (%)
When choosing antiemetic should you consider emetogenic potential of chemotherapy?	62	78.5
When choosing antiemetic did you consider CINV with previous chemotherapy?	59	74.7
When choosing antiemetic did you consider female gender?	42	53.2
When choosing antiemetic did you consider low alcohol use?	29	36.7
When choosing antiemetic did you consider younger age?	34	43
When choosing antiemetic did you consider anxiety?	60	75.9
When choosing antiemetic did you consider history of motion sickness?	42	53.2
When choosing antiemetic do you think there is no risk consideration?	61	77.2
Are you confident in your knowledge of emetogenic potential classification?	29	36.7
Which antiemetic classification system does your hospital use? NCCN, ASCO	57	72.2
With which of antiemetic guidelines are you familiar? NCCN, ASCO	48	60.8
Which antiemetic guideline does your hospital use? NCCN, ASCO	13	16.5
How do you classify AC based chemotherapy when making decision about anti-emetic prophylaxis? NCCN, ASCO	21	26.6

### Practice items

The mean score of oncology nurses’ practice toward guideline recommendation was 0. 4177 about 41.8% of the nurses have scored mean and above for the practice. Out of all respondents, 73.4% of them used 5HT3receptor antagonists to prevent chemotherapy-induced nausea and vomiting that may result from highly emetogenic chemotherapy on day 1(the day of chemotherapy administration) and 83.5% of the respondents use ondansetron most often among the 5HT3 receptor antagonist class. Only 25 (31.6%) of nurses used steroid or 5HT3 receptor antagonists to prevent chemotherapy-induced nausea and vomiting which arises from highly emetogenic chemotherapy after day 2 and beyond, and 20.3% of nurses use only steroid(dexamethasone) to prevent chemotherapy-induced nausea and vomiting which arises from moderately emetogenic chemotherapy after day 2 and beyond but about 76% of participant said their practice regarding anti-emetic agent, they choose for HEC (on day1) consistent with the guideline recommendation.

### Knowledge and associated factors

In binary logistic regression analysis only working department, oncology nursing certified and trained for chemotherapy-induced nausea and vomiting management were found significant determinant factors of knowledge of nurses in oncology setup. As shown in the table below the odds of knowledge regarding chemotherapy-induced nausea and vomiting prophylaxis guideline recommendation working in the outpatient department: [Crude odds ratio (COR)]:3.263; 95% Confidence interval (1.089,9.776), and in inpatient ward [Crude odds ratio (COR)]:5.564;95% CI (1.598,19.375) and chemotherapy administration unit have significance toward CINV management knowledge as shown in Table 3 below. In the multiple logistic regression analysis, there is a change in *p*-value and OR and only some of the variables were found significant. Oncology nursing certified nurses were 1.48-fold more knowledgeable than nurses who were not certified in oncology nursing [Adjusted Odds Ratio (AOR): 1.477; 95% Confidence interval (1.110,1.967). nurses who have trained for chemotherapy-induced nausea and vomiting management were 1.64-fold more aware than those who were not trained [Adjusted Odds Ratio (AOR): 1.638; 95% Confidence interval (1.213,2.212).

**Table 3:** Knowledge of guideline recommendation, practice pattern and, perceived barriers toward Chemotherapy Induced nausea and vomiting prophylaxis guideline adherence. (*n* = 79)

Characteristics	Category	**knowledge items**		***p*-value**	**COR**	***P*-value**	**AOR**
		Poor (%)	good (%)				
Working department	OPD	24%	11.4%	.035*	3.263(1.089,9.776)	0.352	1.102(0.898,1.353)
	Ward	22.78%	6.3%	.007*	5.564(1.598,19.375)	0.054	1.043(.834,1.243)
	Chemotherapy unit	13.9%	21.5%	.015*	1		
Oncology nursing certified	Yes	17.2%	25.31%	.003*	.226(.086,.584)	0.007**	1.477(1.110,1.967)
	No	43.03%	13.9%	.001*	1		1
Trained for CINV management	Yes	13.9%	31.64%	.000*	.071(.023,.218)	.001**	1.638(1.213,2.212)
	No	46.8%	7.59%	.000*	1		1

^*^p value is significant at 0.05

^**^p value is significant at 0.01

### Practice regarding chemotherapy induced nausea and vomiting and associated factors

Binary logistic regression illustrated that gender, working department, having an oncology certificate, having favorable chemotherapy-induced nausea and vomiting management knowledge and training for chemotherapy-induced nausea and vomiting management training were significantly associated with practice in utilization of chemotherapy-induced nausea and vomiting management guideline recommendation and were transported to multivariate logistic regression.

In multivariate logistic regression only having certified in oncology nursing and trained in chemotherapy-induced nausea and vomiting management training remained significantly associated with good chemotherapy-induced nausea and vomiting management guideline utilization practice, however, age, clinical experience in oncology, position in the department and educational level of the participant doesn’t associate with good practice of chemotherapy-induced nausea and vomiting management guideline adherence.

Nurses who work in the oncology ward have 1.102-fold higher good practices compared to other departments [AOR: 1.102; 95% CI (0.898, 1.353). Nurses who have been certified in oncology nursing have 1.477-fold higher good practices than those who haven’t certified [AOR: 1.477; 95% CI as depicted in Table 4 below. Nurses who have favorable knowledge have 0.137-fold higher good practice than nurses who haven’t favorable knowledge [AOR: 0.137; 95% CI (0.045,0.419) (1.110, 1.967) and trained for chemotherapy-induced nausea and vomiting have 1.638-fold higher good practice than nurses who didn’t train for chemotherapy-induced nausea and vomiting management training.

**Table 4:** Knowledge of guideline recommendation, practice pattern and, perceived barriers toward Chemotherapy Induced nausea and vomiting prophylaxis guideline adherence (*n* = 79)

Characteristics	Category	practice items		*p*-value	COR	*P*-value	AOR
		***poor (%)***	***good (%)***				
Gender	Male	25.31%	13.92%	.001*	4.895(1.850,12.951)	0.162	1.210(.926,1.580)
	Female	16.45%	44.30%	.002*	1		
Working department	OPD	20.25%	16.45%	.032*	1	-	1
	Ward	15.18%	13.9%	.016*	4.231(1.314,13.617)	.35	1.102(.898,1.353)
	Chemo administration unit	7.59%	7.84%	.026*	4.000(1.183,13.525)	-	-
Certified in oncology nursing	Yes	11.39%	31.64%	.018*	.315(.120, .823)	0.007**	1.477(1.110, 1.967)
	No	30.37%	26.58%	.615	1		
Trained for CINV management	Yes	7.59%	37.97%	.000*	.119(.041, .346)	.001**	1.638(1.213,2.212
	NO	34.17%	20.25%	.097	1		
knowledge toward CINV	YES	25.31%	35.44%	.000**	.137(.045, .419)	.000**	.137(.045, .419)
	NO	32.9%	6.32%	.250			

^*^p value is significant at 0.05

^**^p value is significant at 0.01

### Barriers toward chemotherapy iinduced nausea and vomiting management

**Fig. 2:**
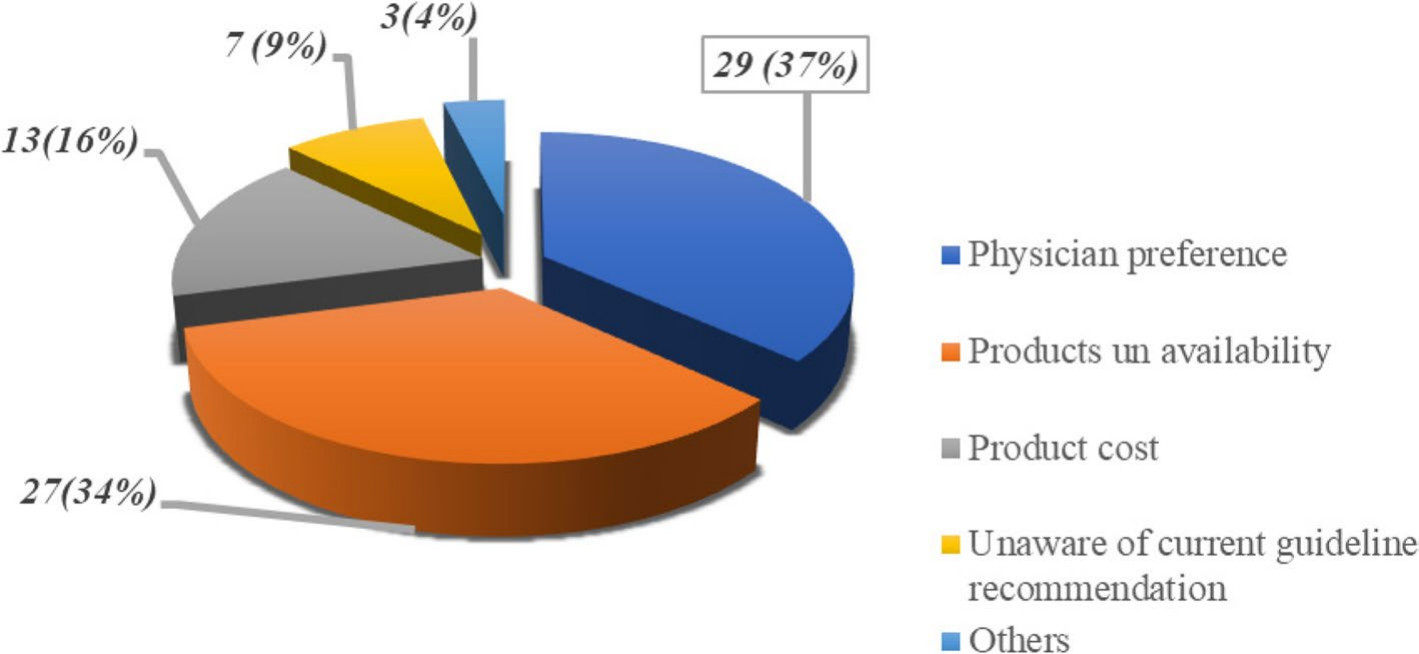
Nurses’ perceived barriers toward chemotherapy induced nausea and vomiting prophylaxis guideline adherence at Addis Ababa, Ethiopia (*n* = 79)

About 98% of the respondents said that there is a specific barrier at our hospital that interfere with or prevent them not to administer the recommended anti-emetics in line with the guideline recommendation. Common barriers identified towards chemotherapy-induced nausea and vomiting management as shown in Fig. 2 below are Physician interference (36.7%), products un availability (34.2%) and products cost (16.5%) were the three top barriers of nurses.

Controlling chemotherapy-induced nausea and vomiting in the delayed phase (day 2 and beyond) (29%), controlling chemotherapy-induced nausea and vomiting in the acute phase (26.6%), and lack of access to modern antiemetics to prevent and manage Chemotherapy Induced Nausea and vomiting are the major challenges of nurses on their career.

## Discussion

The current study reveals the knowledge, practice, and perceived barriers of nurses regarding chemotherapy-induced nausea and vomiting at selected public hospitals in Addis Ababa. Of the total of 81 oncology nurses working in both hospitals, 79 of them participated in this study, with a response rate of 96%. About 60.8% of the study participants responded correctly to the knowledge assessment, but 63% of the nurses are not confident in their knowledge of emetogenic classification of different types of chemotherapy and chemotherapy-induced nausea and vomiting management. This result is supported by research done among Registered nurses who were administered chemotherapy to cancer patients in Australia, China, Hong Kong, and Nine Latin American countries who completed a self-reported survey. This study found more than one-third of participants are not following the standard guidelines for chemotherapy-induced nausea and vomiting, but they are using their own knowledge in practice [16, 17].

The finding of this study is less than the study conducted in the USA which reveals that Nurses in the outpatient oncology setting stated significantly greater confidence in their knowledge; 75% of those in outpatient settings said they were confident or very confident, compared to 57% of those in inpatient and 47% in the other settings (*p* = 0.002)15. This discrepancy might be due to cultural, and socio-demographic differences, differences in work experience, study time gaps, and study setting differences. Being small in number and working closely with oncology physicians could also be another reason for good overall nurses’ knowledge [15].

Most participants (96.5%) agreed that chemotherapy-induced nausea and chemotherapy-induced vomiting would consider separately (79%), but only 35% were confident in their ability to manage chemotherapy-induced nausea.

The odds of knowledge regarding chemotherapy-induced nausea and vomiting prophylaxis guideline recommendation working in OPD [COR:3.263; 95% CI (1.089,9.776), and in inpatient ward [COR:5.564;95% CI (1.598,19.375) and chemotherapy administration unit have significance toward CINV management knowledge. This is consistent with the study done in the USA which reveals that Nurses in the outpatient setting stated significantly greater confidence in their knowledge; 75% of those in outpatient settings said they were confident or very confident, compared to 57% of those in inpatient and 47% in the other settings (*p* = 0.002) [14, 15].

About 60.8% of the participant were familiar with the national comprehensive cancer center; NK-1:Neurokinin-1guideline but only 16.5% of the participant knows which guideline was used in their institution in contrast another Study was done in the USA showed that nurses were most familiar with the national comprehensive cancer network (73%) and American society of clinical oncology (48%) antiemetic guidelines, the reason behind that was American Society of Clinical Oncology guideline was currently not used in our set up rather black lion and St. Paul’s hospital uses national comprehensive cancer center; NK-1:Neurokinin-1 guideline [18, 20].

This study shows Only 20% of the participant uses steroid(dexamethasone) to prevent Chemotherapy Induced nausea and vomiting for Moderate emetogenic effect chemotherapy on day 2 and beyond. Another study done in 16 European countries shows key discrepancies between antiemetic use and guideline recommendations were: i) underutilization of NK1RAs, 5-HT3RAs, and a steroid on day 1 in the HEC setting and ii) high use of 5-HT3RAs during days 2–5 when guidelines recommend a steroid [20].

The finding of this study showed that certification in oncology nursing was significantly associated with knowledge of chemotherapy-induced nausea and vomiting prophylaxis guideline (*P*-value < 0.05, 95% CI). Participants who were certified in oncology nursing were 1.48 times more likely knowledgeable than those nurses who were not certified in oncology nursing [AOR: 1.477; 95% CI (1.110,1.967). this might be due to most of the participant who has oncology nursing certificates have also more experience in the care of cancer patient with chemotherapy which might, in turn, improve their knowledge of chemotherapy-induced nausea and vomiting prophylaxis guideline recommendation. The result of this study is in line with the study carried out in the USA which revealed that there was a significant and strong correlation between chemotherapy-induced nausea and vomiting prophylaxis guideline recommendation knowledge and is certified in oncology nursing (*P* < 0.011) [16]. This implies that knowledge of chemotherapy-induced nausea and vomiting prophylaxis guidelines can be improved if nurses were trained and certified in oncology nursing in the study area nurses assigned in the oncology unit were comprehensive nurses, not oncology nurses. Physician interference (36.7%), products un availability (34.2%), and product cost (16.5%) were the three top barriers to nurses. Supported by a study In the USA showed that, the predominant barrier interfering with guideline-recommended antiemetic prophylaxis was reported as physician preference 71% [14, 19].

### Limitation of the study

The limitation of this study noted that a survey and a single quantitative approach were applied. A quantitative approach may not explore detailed and hidden information. It is recommended to apply mixed methods that can be explored by nurses’ Knowledge, practice, and perceived barriers towards chemotherapy-induced nausea and vomiting in prophylaxis guideline adherence in oncology units. And also, a cross-sectional study design with a small sample size limits generalized.

## Conclusion and recommendations

### Conclusion

Significant gaps exist in nurses assigned and working in the oncology unit of adherence to chemotherapy-induced nausea and vomiting prophylaxis guidelines in the two study public hospitals of Adds Ababa.

Nurses are not confident in their knowledge of emetogenic classification of different types of chemotherapy Working department, being certified in oncology, and getting chemotherapy-induced nausea and vomiting training have a significant association with good knowledge of chemotherapy-induced nausea and vomiting management guidelines and statistically significant association.

Physician preference, product unavailability, and cost of the anti-emetic drug were the major barriers to nurses not to adhere chemotherapy-induced nausea and vomiting guideline recommendation and un availability of potent anti-emetics were the challenge for nurses in oncology setup.

### Recommendations

- Oncology units of the study hospitals (Tikur Anbesa Specialized Hospital and Saint Paul Hospital Millennium Medical College) should consider using the recent antiemetic guideline to improve the management of chemotherapy-induced nausea and vomiting.
- The nurse manager should consider planning and providing in-service training on the use of chemotherapy-induced nausea and vomiting in prophylaxis guideline adherence for all nurses working in the oncology unit.
- The nursing management team encourages and must create a conducive environment for nurses to avoid the barriers that alter the nurses not to work in line with the recommended guideline.
- The nurse must be competent enough in their practice and knowledge by updating their skills and knowledge to apply the recommended guideline.

## Data Availability

All relevant data are included with in the manuscript document. If it is necessary, it is possible to contact the corresponding author to get additional materials.

## Abbreviations

AAU: Addis Ababa University
ASCO: American Society of Clinical Oncology
AUC: Area under curve
CINV: Chemotherapy Induced Nausea and Vomiting
GCCP: Guideline Inconsistent Chemotherapy Induced nausea and vomiting Prophylaxis
GI: Gastrointestinal
HEC: High emetogenic effect chemotherapy
MEC: Moderate emetogenic effect chemotherapy
IRB: Institute of review board
MASCC: Multinational association of supportive care in cancer
N&V: Nausea and Vomiting
NCCN: National comprehensive cancer center
NK-1: Neurokinin-1
NK-1RA: Nurokinin-1 receptor antagonist
5HT-3: Serotonin5-hydroxytrypitamin
5HT-3R: Serotonin-5 hydroxytrypitamin receptor antagonist
SPSS: Statistical software for social science
TASH: Tikur Anbessa Referral hospital
